# Responsibility as an Obstacle to Good Policy: The Case of Lifestyle Related Disease

**DOI:** 10.1007/s11673-018-9860-y

**Published:** 2018-06-06

**Authors:** Neil Levy

**Affiliations:** 1 Department of Philosophy, Monash University, Melbourne, VIC 3800 Australia; 2 Uehiro Centre for Practical Ethics, University of Oxford, Oxford, OX1 1PT UK

**Keywords:** Responsibility, Lifestyle disease, Obesity, Policy

## Abstract

There is a lively debate over who is to blame for the harms arising from unhealthy behaviours, like overeating and excessive drinking. In this paper, I argue that given how demanding the conditions required for moral responsibility actually are, we cannot be highly confident that anyone is ever morally responsible. I also adduce evidence that holding people responsible for their unhealthy behaviours has costs: it undermines public support for the measures that are likely to have the most impact on these harms. I claim that these two facts—the fact that we cannot be highly confident that anyone is morally responsible and the fact that holding people responsible for their unhealthy behaviours has costs—interact. Together they give us a powerful reason for believing, or acting as if we believed, that ordinary people are not in fact responsible for their unhealthy behaviours.

People are *causally* responsible for many of their own problems. Indeed, in the arena of healthcare, up to 40 per cent of premature deaths are preventable by changes to lifestyle (Yoon et al. 2014). The biggest causes of lifestyle-related morbidity and mortality are behaviours which are widely known to be unhealthy (smoking, unhealthy diets, lack of exercise, and excessive drinking). These facts make the following inference *prima facie* plausible: if agents are causally responsible for their ill-health, and the causes are voluntary behaviours they know to be linked to ill-health, they are also *morally* responsible for their ill-health. That conclusion need not be taken to entail that they are not entitled to healthcare, say. However, it (plausibly) has some practical import. It may, for instance, bear on how their entitlements are to be weighed against those of others who are not responsible for their ill-health, when it comes to the allocation of scarce resources.

There is ongoing debate about the truth and the usefulness of holding individuals responsible for unhealthy behaviours (Wikler 2004; Brown 2013; Albertsen 2015). Here I do not aim to contribute directly to these debates. I will not engage in any detail with arguments over the extent to which ordinary people or some class of people (say addicts) satisfy the conditions laid down by standard accounts of moral responsibility. Instead, I will argue that regardless of how well individuals satisfy these conditions, there are strong reasons to resist holding them responsible for their ill-health. These reasons are partially epistemic and partially pragmatic. Epistemically, I shall suggest, the evidence available suggests that it is *reasonable* to doubt that anyone is responsible for their voluntary behaviours. Pragmatically, I will argue that holding people morally responsible for their ill-health is an obstacle to tackling the challenge of lifestyle related morbidity and mortality. These two factors interact: when it is reasonable to hold that *p* or that not-*p*, we may refer to pragmatic factors in order to decide whether we ought to believe, or to act as if we believe, that *p*.

In the first section of this paper, I will briefly outline the *prima facie* case for holding people responsible for their own ill-health. In the next section, I will turn to worries about moral responsibility generally. I will suggest that the conditions that must be satisfied for agents to be responsible are demanding, and that we lack grounds to be very confident that they are satisfied. I do not suggest that we should think that we are not morally responsible, or even that we should withhold judgement on the question. Rather, I will suggest that in the context of lifestyle related problems in particular, we should refrain from attributing responsibility, in light of the evidence (presented in the subsequent section of the paper) that such attribution mitigates against effective policy responses to lifestyle-related diseases.

The aims of this paper are therefore restricted to examining the implications of sceptical arguments against moral responsibility for one particular question. Indeed, its scope is narrower still. I do not pretend to offer either actual policy advice or even to offer conclusive considerations that favour one kind of advice. Rather, this paper is an exercise in ground-clearing. I aim to show that there are strong reasons to avoid holding people responsible for their lifestyle-related problems. The conclusion is negative and leaves a great deal of scope for positive proposals on actual policy. I do not apologize for this narrowness: effective policy development requires the expertise of many different kinds of people with different kinds of disciplinary backgrounds. Offering purely negative advice can be an important contribution to such development.

## The Apparent Moral Responsibility of the Unhealthy

There is a prima facie case for holding those of us who repeatedly engage in unhealthy behaviours morally responsible for resulting ill-health. On standard accounts, agents are morally responsible for their actions and omissions when they satisfy two conditions: a control condition and an epistemic condition. Perhaps due to the influence of the free will debate, which overlaps considerably with the moral responsibility debate, the control condition has received most of the attention and sophisticated models of the condition have been developed.

On perhaps the single most influential (semi)-compatibilist account of control, an agent exercises control over her behaviour if it is the product of a reasons-responsive mechanism of her own (Fischer and Ravizza 1998).^1^ To say that the mechanism is the agent’s own is to say that it has the right kind of history (I set this condition aside here). Control itself consists in moderate reasons-responsiveness, where a mechanism is moderately reasons-responsive if it is capable of tracking reasons and also of causing the agent to act differently if the right reasons are presented. There is a great deal of debate over the details of this and related accounts, but we can ignore this debate in this context. The important fact to recognize is that very many people satisfy these conditions with regard to their unhealthy behaviours.

There are, indeed, problem cases regarding which it is very unclear whether the agents satisfy the standard conditions for being responsible. Some lifestyle-related health problems begin in childhood (for instance, there has been rapid growth recently in obesity and type 2 diabetes among children), and children may not satisfy either control or epistemic conditions on moral responsibility. Even once they become responsible adults, it may be very difficult to address these problems, which at least mitigates responsibility for their persistence. Addiction, which obviously plays a role in the development of some severe health problems (lung cancer, heart disease, liver failure, and so on), also raises significant challenges. Do addicts exercise sufficient control to count as morally responsible for their drug and alcohol intake? Might they be morally responsible for being addicted (in virtue of having voluntarily begun to consume, prior to the development of their addiction) and therefore derivatively responsible for the harms they undergo? These are difficult questions, on which there is no consensus in the literature. But it seems plain that very many people satisfy the epistemic condition and the control condition with regard to many of the behaviours that cause significant harms. Many of us eat too much, despite knowing that we do so. Many of us exercise too little and know that we do so. We are responsive to reasons against doing so—not only do we recognize that we shouldn’t engage in these behaviours, we are capable of reacting to reasons to do otherwise (if the price of chocolate quadrupled, we might stop eating it). Since we satisfy these conditions with regard to these behaviours (and many more), it seems there is a strong case for holding us morally responsible for the resulting harms.

One way to put the point is as follows. There is a plausible account (or set of accounts) of the properties that make agents morally responsible. We satisfy the conditions laid down by this account for many of our harmful actions. We must therefore accept that *either* we are responsible for the resulting harms *or* that the account is too demanding. But the latter option is not attractive, because the account is very minimal and *un*demanding. If it is too demanding nevertheless, it seems that no one, or very few people, are ever morally responsible for anything.

Despite the fact that most people find the possibility that we lack moral responsibility unpalatable, I think this is a possibility we should take seriously. Not only are there are powerful arguments for responsibility scepticism, there are also reasons to worry that those people who reject scepticism are too optimistic in doing so. Many participants in debates over rival accounts of moral responsibility advance demanding conditions for its existence, and these are conditions that may not be satisfied. I do not aim to argue that we are not morally responsible for our actions and omissions. Rather, I have a more modest goal in mind: showing that scepticism deserves to be taken seriously in the context of lifestyle-related problems. It remains unclear whether we can do without moral responsibility attributions, both in interpersonal life and in the criminal justice context (Strawson 1962; Pereboom 2014; Caruso forthcoming). Because it remains unclear, we may be “stuck” with such attributions, whether they are justified or not. But matters are very different in the context with which I’m concerned here, because there is evidence that holding people responsible has, all things considered, costs in this context. If scepticism deserves to be taken seriously, and (as I will argue in the second half of this paper) holding people responsible is likely to have costs, we have reason to err on the side of refusing to hold responsible.

## Sceptical Worries

The moral responsibility debate, in its standard form, is familiar to most philosophers. It concerns whether free will (a necessary condition of moral responsibility on most views) is compatible with determinism. Most philosophers are compatibilists, holding that free will does not require indeterminism, but there is a significant minority of philosophers who are incompatibilists (Bourget and Chalmers 2014), and a substantial proportion of ordinary people are libertarians, holding that free will is incompatible with determinism but also maintaining that determinism is false (Nichols and Knobe 2007; Nahmias 2011). More intriguingly, after a long period of retreat in the 1960s and 1970s, incompatibilism may be the majority view among those people who have thought hardest about the problem: those who actually work on it (Van Inwagen 2004). Of course, there may be selection effects in play (perhaps people who worry whether we have free will are more likely to work on it, and it is therefore because they are incompatibilists that they become free will specialists, rather than their becoming incompatibilists because they are free will specialists). Nevertheless, it seems that we have grounds for taking incompatibilism seriously. And incompatibilism is a demanding view. If incompatibilism is true (as many of the people who’ve thought most deeply about the issues believe) we could easily fail to be morally responsible for our actions.

If incompatibilism is true, we are morally responsible for our actions only if the world is indeterministic. Now, many physicists believe that the world is indeterministic (because many accept an indeterministic interpretation of quantum mechanics). It might therefore seem that we can relax on that front: if moral responsibility requires indeterminism, we are probably sometimes morally responsible for our actions. But relaxing too quickly would be a mistake. While arguments for incompatibilism are typically arguments designed to show that if determinism is true, then we lack control over (or fail to be the source of) our actions, positive indeterministic accounts of free will (that is, libertarian accounts) make it clear that what is needed is not just the falsity of determinism but indeterminism of the right *kind* in the right *places*. And we can’t safely assume that either condition is satisfied.

For example, if indeterminism is to be relevant to our freedom it had better be located internal to decision-making. That almost certainly means that our brains must be indeterministic. But there are questions whether the indeterminism supposed to be entailed by quantum mechanics can affect events on the scale of neural firings—whether quantum level indeterminacies can make a difference to relatively large (and wet) systems like the brain (Hodgson 2012). Further, it is not enough that neural events are indeterministic. Libertarianism faces the notorious problem of luck—don’t indeterministic events, over which we have no control, make us less, rather than more free, by rendering our actions subject to luck?—and solving that problem requires the postulation of the right *kind* of indeterminism in the right *place* and to the right *extent*. Indeterminism sufficient to open up alternatives has to occur when and as the result of agents trying to do two incompatible things at once, according to Robert Kane (1996), for instance, or it has somehow to dovetail with our (supposed) power of agent causation, according to Timothy O’Connor (2000).

Here is not the place to go into the details of these theories and assess their plausibility. The point I want to make is just this: these theories set down demanding conditions for us having the kind of free will required for moral responsibility, and while their proponents take them to be satisfied, they are able to adduce very little evidence that they are. These facts entail that we find ourselves in an interesting dialectical position.

Almost everyone believes that agents are often morally responsible for their behaviours. Only 12.2 per cent of philosophers accept or lean toward thinking we lack free will (Bourget and Chalmers 2014), and the proportion of laypeople who hold that view may be very much smaller. But there is nothing like a consensus on sufficient conditions for free will. Moreover, many people think that these conditions are quite demanding. If we do not know whether sufficient conditions for free will (again, understood here, as is usual, as a necessary condition for moral responsibility) are satisfied, then it seems that we do not know whether agents are indeed morally responsible.

It is true that compatibilists often deny that the sufficient conditions for moral responsibility are demanding. While once some compatibilists held that determinism was not merely compatible with, but actually required for, moral responsibility, most are now “supercompatibilists,” holding that moral responsibility is compatible with determinism *and* indeterminism (Vargas 2012). But it is a mistake to think that supercompatibilists do not set down demanding conditions for moral responsibility. Consider the luck objection: the standard objection to libertarian theories that indeterminism in the causal route from cognition to behaviour (regarded by libertarians as a necessary condition of moral responsibility) makes our behaviour unacceptably subject to luck (Mele 2006). Given that many people think that neural processes are indeterministic, supercompatibilists must face the luck objection along with libertarians. It is very far from clear that there is a satisfactory response to the luck objection, so once again we should think that our confidence that agents are often morally responsible is misplaced.

Further, there are threats to moral responsibility other than those stemming from the luck objection that the supercompatibilist must confront. Recent philosophers have suggested that naturalism (Waller 2011), a kind of luck that is a feature of our world whether it is deterministic or indeterministic (Levy 2011), an unsatisfiable need of self-creation (Strawson 1994), or contingent facts about how the brain happens to work (Caruso 2012) entail that we lack the freedom needed for moral responsibility. While compatibilists have dismissed some of these requirements as hyperbolic—metaphysical megalomania, in Fischer’s (2006) memorable phrase—most seem to constitute serious challenges that cannot be dismissed in this way. Again, there are responses to each challenge available, at least some of which are plausible. But it also seems plausible that no one can confidently conclude that we know that every one of these challenges fails. Given the range of challenges to the existence of the kind of freedom required for moral responsibility, I think we find ourselves in the following situation: while almost all of us are confident that we and our fellow agents are routinely morally responsible for actions, we do not know that the necessary conditions for this kind of freedom obtain. That should lower our confidence that we have this kind of moral responsibility.

In a number of papers, Benjamin Vilhauer (2009, 2012) has argued for an analogous conclusion on parallel grounds. Vilhauer argues that anyone who takes the possibility of free will scepticism seriously, where taking free will scepticism seriously is thinking that such scepticism is intellectually respectable enough to be worth engaging with, is committed to thinking that harsh punishment is unjustified. Vilhauer argues that taking free will scepticism has practical implications that other kinds of scepticisms (scepticism about other minds or about induction, for instance) do not have. The reason is that free will scepticism has direct implications for one of our central moral principles: that innocent persons should not be unjustifiably harmed. Whereas other scepticisms might threaten this principle itself (by undermining our warrant for knowledge more generally, say, or our grounds for concern about other agents), free will scepticism leaves it untouched. Taking free will scepticism seriously therefore leaves us with stronger epistemic reasons to be committed to this moral principle than to thinking that those who might be harmed by punishment deserve these harms. My argument is clearly parallel to Vilhauer’s. There are two differences worth highlighting, however.

First, my argument is epistemic and pragmatic—turning on the confidence we are justified in having in whether sufficient conditions on moral responsibility are ever satisfied, and on the costs of being wrong about this—rather than addressing itself to those who take free will scepticism seriously. It therefore has a wider scope than Vilhauer’s so far as its audience is concerned. But it has a narrower scope in another way. Vilhauer is concerned with moral responsibility generally: I am concerned with responsibility only in the context of health-related behaviour. Focusing on this context is not an unprincipled limitation. There are a number of important differences between the broader questions concerning moral responsibility that are the focus of the traditional debate and the question of individual responsibility for ill-health. First, the moral responsibility debate is one that raises passions, because the stakes are so high. It concerns the responsibility of people who have done awful things and on how we best protect ourselves from them. The costs of implementing policies based on false views are potentially catastrophic in this domain. Second, the broad debate is a perennial one in philosophy and not one that is going to be resolved soon (if ever). Third, and most importantly, our epistemic situation is different with regard to the broad and narrow debates. While there is plentiful evidence that our current criminal justice system imposes harms on people, it is extremely hard to assess the balance of costs and benefits with regard to moral responsibility generally. Holding people responsible for their own ill-health is crucially different: the evidence suggests that the costs of doing so outweighs any benefits.

Thus, while the preceding discussion has concerned moral responsibility, broadly construed, it has very different implications for our responsibility for our own health than it has for (say) the criminal justice system. What I take myself to have succeeded in showing is that our confidence that we are morally responsible (in all contexts) should be lower than we tend to think. This fact—the fact that our confidence that agents are morally responsible should be lower than we tend to think—has particular implications for the kinds of contexts with which I am concerned in this paper. In these contexts, I will suggest that the benefits of holding responsible are likely smaller than the costs, and the costs of wrongly holding responsible are likely significantly greater than the costs of wrongly failing to hold responsible. In the next section, I will attempt to show that there is evidence that holding people responsible for their own health has all things considered bad consequences for policy and outcomes.

## The Harms of Holding Responsible

We might want to hold people *morally* responsible for the harms constituted or caused by their own health outcomes in a variety of contexts. We might want to allocate scarce resources (say organs for transplant) to people in a way that is sensitive to their responsibility, for instance. We might think that an alcoholic has a less weighty claim on a liver for transplant than another patient, so that other things being equal the “innocent” patient ought to be prioritized. We might want to limit access to public support for addicts, in the belief that they do not deserve such support when some proportion of it might be spent on drugs. Or we might think that each individual is responsible for his or her consumption decisions and oppose initiatives that require businesses to prompt them to make better choices (say limitations on serving sizes, or nutritional information on menus, or the taxation of certain products to ensure that they are relatively more expensive) or even to enforce such choices (say limiting the amount of sugar in beverages) on these grounds. If agents are morally responsible for their choices and the consequences of their choices, these initiatives are less likely to be justifiable because they impose costs on people and because they limit their choices or even infringe on their autonomy. If on the other hand agents are not morally responsible for their choices and for the consequences of their choices, then the threshold for justifying these initiatives is lower.

As a matter of fact, worries about responsibility and about closely related questions like autonomy have been central to opposition to initiatives like these. Public opinion tends to regard obesity, for example, as a matter of personal (or parental) responsibility (Hardus et al. 2003; Covic, Roufeil, and Dziurawiec 2007; Lund, Sandoe, and Lassen 2011). Parents are held to be much more strongly responsible for the obesity of their children than are institutions, governments, or corporations (Wolfson et al. 2015). More interestingly and worryingly, Wolfson et al. (2015) found that those individuals who attributed more responsibility to parents than to “external” agents (schools, government, corporations) showed significantly less support for allowing individuals to sue food and beverage makers for weight gain associated with their products and significantly less support for a tax on sugary food, compared to individuals who attributed more responsibility to external agents. Those who blamed parents also exhibited weaker support for a ban on advertising unhealthy foods during television programmes directed at children.

Wolfson et al. (2015) note that those who held parents more responsible than external agents did not oppose all proposals that are aimed at improving health by changing institutions. For instance, they supported proposals to mandate physical activity in schools. One way to read this pattern of results is as follows: respondents who attributed more responsibility to individuals rather than to external agents supported policies directed at the latter so long as these policies are not *punitive*. A policy is punitive when it imposes all-things considered costs on the agent targeted by it, where something is an all-things considered cost when in the absence of measures to impose it, the agent might achieve her goals without paying it. Clearly, being sued (successfully) imposes costs on corporations; less clearly, but plausibly enough, being prevented from advertising also imposes all-things-considered costs on them. But requiring exercise in schools does not impose an all-things-considered cost on the schools. Of course, teachers must oversee the exercise and space must be provided for it. These things cost money. But this may not count as the imposition of an all-things-considered cost to the school, because it is not an impost that it must take into account *additionally* to those it must expend in pursuit of its goals. If you think that contributing to the healthy development of children is a school’s goal, then this is not a cost. It is not something that must be borne as the cost of achieving its goals; it is a constituent of achieving its goals. The finding that attributing responsibility to individuals is consistent with some limited proposals that target institutions is therefore consistent with thinking that this attribution is an obstacle to the development of social policies that address unhealthy behaviours. Agents are disposed to think that punitive measures—those that impose all-things-considered costs—may only be directed at individuals and institutions that are responsible for harms.

Certainly, corporations and their defenders appear to believe that a focus on the (putative) responsibility of individuals for their consumption decisions will allow them to avoid bearing burdens associated with addressing unhealthy behaviours. There is evidence that the tobacco industry deliberately used the rhetoric of personal responsibility to deflect calls for regulation (Mejia et al. 2014). The same rhetoric was adopted by the food and beverage industry, in a largely successful attempt to avoid restrictions on consumption or special taxes on their products (Kersh and Morone 2002; Brownell et al. 2010). These industries themselves apparently believe that if they can increase the perception that individuals are responsible, they thereby bring it about that they are less likely to be held responsible themselves.

Moral responsibility is not zero-sum. Suppose I know that my enemy is a recovering alcoholic and I contrive for him to be in a situation in which it is difficult for him to resist the temptation of alcohol. Plausibly, I am responsible for some of any resulting harms, but that does not reduce his responsibility for them.^2^ Or consider two independent assassins, both of whom shoot the mayor. Either bullet would have been sufficient to kill her; accordingly, each is fully responsible for her death. Responsibility can be shared without being reduced. However, the evidence above suggests that as *a matter of fact*, people often inappropriately treat responsibility as tending toward being zero sum. That is, we tend to diminish (though perhaps not to eliminate) the responsibility of one agent or set of agents when we identify another agent as responsible for the same event.

A likely explanation for why some agents are more salient to us and more apt to be ascribed responsibility than others is our naïve theory of causality. It is a commonplace that some of the events and states that are necessary for the occurrence of an event are much less likely to be ascribed the status of causes than others. A hackneyed example: we say that the lightning caused the fire, even if we recognize that there were many background conditions that had to prevail for the fire to occur. The presence of oxygen is very unlikely to be identified as a cause, for instance. As Hitchcock and Knobe (2009) have suggested, we identify *the* cause by reference to norms: the cause of an event is an event or state of affairs that deviates from the background conditions we regard as normal (so the ongoing drought is more likely to be considered the cause of the fire if we regard the lack of rainfall as a deviation from the norm, and lightning as an expectable occurrence). Because we take the environment which is pervasively shaped by corporations and the prevailing laws as a background condition, we tend to regard parents and individuals who act within it as *the* causes of unhealthy behaviours and downplay the role of those who shape the landscape in which they act.

Our intuitions about responsibility are downstream from our theories about causation: we attribute responsibility by asking first who caused an event. That being so, given that we are liable to attribute responsibility to actors who are salient to us, and not to those who structure the environment in which they act, and given our tendency to regard responsibility as tending toward zero sum, we are likely to overestimate individual responsibility for unhealthy behaviours and underestimate institutional or corporate responsibility.^3^ That is a problem, for two reasons.

First, there is a strong case for holding corporations and governments (at least) partially responsible for the harms in question. They (or individuals with very significant decision making powers) have not merely allowed conditions to prevail in which it is difficult for individuals to make healthy choices; they have actively campaigned to prevent changes to these conditions when these changes would affect their profits.^4^ When agents deliberately act in a way that they know will likely bring about harms, there is a strong prima facie case for holding them partially responsible for these harms, even *if* these harms arise only with the cooperation of other agents who are themselves (at least partially) responsible (compare: I know my neighbour is a vicious person, and wanting to see him cause mayhem, I leave a knife where I know he will find it. He may be entirely responsible for any damage he causes, but I am due some blame too, it seems).

Second, even if we set aside the question of whether corporations (or the individuals who constitute them) are responsible for the harms in question, there is a very strong case for holding that we should respond to unhealthy problems by treating them as social problems that are best dealt with by altering policies. Admittedly, actual trials of sugar taxes have so far produced equivocal results (Silver et al. 2017; Silano and Agostoni 2017). However, we have excellent reason to be confident that changes to the environment in which people make consumption decisions can be effective in reducing obesity (and, by implication, in responding to other lifestyle related diseases). The reason is this: obesity has become a major problem worldwide very recently, over timescales far too short to be explicable by changes in gene frequencies in human populations. This fact entails the high plausibility of explanations for the relatively sudden and very rapid growth of the problem which turn on the environment in which we find ourselves, such as the availability of cheap, tempting, and energy dense foods, and factors which affect how much we eat and how sedentary we are. While it remains an open question how we can best alter the environment to combat the problem (and at lowest costs, in terms of restriction of individual liberties and unfair distribution of burdens), there can be little doubt that such alterations can be effective.

As we have seen, attributing responsibility to individuals for their unhealthy behaviours, or to parents for the behaviours of their children, lowers support for policies that address the conditions in which these behaviours unfold (such as the regulation of advertising aimed at children or the imposition of sugar taxes), insofar as these policies are seen as punitive. Even if corporations are not responsible for the unhealthy behaviours of individuals, these policies may be justifiable. In particular, they may be justifiable if *no one* is responsible for these behaviours, and no one therefore *deserves* to suffer the associated burdens. If that’s the case, then we should decide how the burdens are distributed on consequentialist grounds, and that calculus is likely to yield the result that the policy changes are justified.

## The Interaction of Responsibility Scepticism and Pragmatic Considerations

In the first part of this paper, I argued that high confidence that anyone is morally responsible for their actions is not justified. I noted, however, that given the potential costs of rejecting moral responsibility in the criminal justice system, and the fact that in this domain we may be required to act as if people are responsible, whether they are or not, it is far from clear that anything follows from this conclusion as far as policy (or, indeed, our individual responses to wrongdoers) is concerned. However, the conclusion might be action guiding with regard to responsibility in restricted spheres, such as the sphere of responsibility for ill-health. When we have good evidence that the costs of holding people responsible in these spheres are high (and the costs of being wrong about that are relatively low), our low confidence that agents *are* responsible justifies taking action.

The evidence reviewed above suggests that blaming individuals tends to reduce public support for measures designed to change behaviours, thereby reducing the likelihood that we respond appropriately. Our response to this fact should be modulated by our lack of high confidence that individuals are responsible for their choices. There are two reasons why evidence concerning the costs and benefits of holding individuals responsible should have different implications for different spheres of behaviour. The first is epistemic; the second pragmatic.

First, our acceptance of claims is and should be sensitive to our confidence in them.^5^ Inspector Maigret has better reason to arrest Lapointe if he is highly confident that Lapointe is the murderer than if he merely thinks that Lapointe is more likely than not to be the murderer. Second, our acceptance of claims should also be sensitive to the stakes. Maigret may permissibly act on his belief that his pipe is his pocket without checking if the costs of being mistaken are low (and especially if the costs fall largely on him). Similarly, if we are not very confident that individuals are responsible for their choices, we have better reason to refrain from overtly attributing responsibility to them than if we are highly confident. Our reason to refrain from attributing responsibility is stronger still if we recognize that our doing so has costs. In this case, the costs are high, given the number of early deaths that might be prevented by changing people’s behaviour.

Thus, we have both epistemic and pragmatic reasons to take our low confidence that the conditions for moral responsibility are ever satisfied seriously in the sphere of responsibility for health outcomes. Other things being equal, our acceptance of claims ought to be sensitive to our degree of confidence in them. Other things being equal, our acceptance of claims should be sensitive to the stakes of being wrong. Given the evidence that our confidence should not be high, and the evidence that the costs of wrongly holding people responsible, in this domain, are likely to be greater than the costs of wrongly exculpating people from responsibility, we have good reason for refraining from attributing moral responsibility to individuals for their own ill-health.^6^

## Conclusion

There are few or no genuinely decisive arguments in ethics. Live debates are live for a reason: there genuinely are powerful arguments on both (or all) sides, and none can reply to objections in a way that compels agreement. I do not take myself to have succeeded where almost everyone else has failed. I certainly do not take myself to have shown that ordinary people are not responsible for their unhealthy behaviours. Nor can I claim to have shown that the costs of holding them responsible outweigh the benefits.

I claim nevertheless to have provided a strong case for holding that we ought not to hold individuals responsible for their unhealthy behaviours. Given how demanding are the conditions that many people (including a majority of those who have thought most deeply about these issues) hold are necessary for moral responsibility, we cannot be highly confident that anyone is morally responsible for their actions. While that it is a conclusion, arguably, that ought not to make us rethink the criminal justice system (perhaps the latter can be justified on consequentialist grounds), it provides a strong reason for refraining from holding people responsible for unhealthy behaviours. If there is good reason to think that holding people morally responsible for their unhealthy behaviours has costs, then our lowered confidence that they *are* responsible should lead us to believing, or acting as if we believed, that they are not responsible.

Perhaps I have underestimated how powerful the arguments in favour of moral responsibility are. Perhaps the costs, in this context, are lower than I think. Or perhaps there are countervailing reasons for retaining responsibility for unhealthy behaviours (we might worry that holding responsible plays a role in developing and sustaining agency, for example). Nevertheless, given the enormous costs of lifestyle related disease, I think the case is persuasive enough to motivate policy.

## Footnotes

1 Compatibilism is the thesis that free will (and/or moral responsibility) is compatible with the causal determinism of all our actions; causal determinism, in turn, is (very roughly) the thesis that every event is caused by prior events, and given the prevailing laws of nature, no other event than the one that was actually caused could occur. It is important to note that incompatibilists often accept something very like the compatibilist account of control sketched in the main text. They hold that such control is a necessary but not sufficient condition of moral responsibility. The account sketched is a *semi-*compatibilist account because it holds that agents may be morally responsible in a deterministic world, whether or not free will is compatible with causal determinism.

2 To see this, consider an analogous case in which he just happens to find himself in a situation in which it is difficult for him to resist the temptation. Since the context in which he finds himself is relevantly identical and his capacities in the context are relevantly identical, he is just as responsible in this case as in the last. So the fact that I am responsible for his being in a context, and to some degree responsible for the resulting harms that I foresee, can’t make a difference to *his* responsibility

3 Some philosophers deny that there is any such thing as corporate or collective responsibility or hold that such responsibility is extremely rare (e.g. Sverdlik 1987; Corlett 2001). However, these debates need not detain us, for two reasons. First, even if these critics of collective responsibility are right (the view is rapidly becoming less popular), much the same set of issues would remain. The only thing that would change would be that phrases like “the responsibility of corporations” would have to be interpreted as shorthand for whatever claim the critics allege they can be reduced to (perhaps “the responsibility of senior managers”). Second, no one denies that there is *some* sense in which it may be apt to hold corporations responsible. We may, for instance, impose restrictions on them due to their causal roles. Responsibility sceptics do not deny the aptness of forward-looking responsibility ascriptions, justified on consequentialist grounds, and therefore may accept such ascriptions with regard to corporations consistent with their scepticism about moral responsibility more generally.

4 For evidence that the conditions in question (ranging from the placement of advertisements to the structure of malls and even streetscapes) makes it difficult for individuals to make better choices, see Levy 2012.

5 I mean “acceptance” here to be neutral between *believing* a claim and *acting on* the claim. There is ongoing debate over whether we can exercise direct control over our beliefs. While everyone accepts that it is not possible to believe just any proposition at will, some philosophers claim that there are circumstances in which we can decide to believe a proposition; others deny that beliefs are under direct control at all (see Levy 2007; Levy and Mandelbaum 2014 for discussion). I do not take a stand on this question here. Whether or not we can exercise direct control over our beliefs, we can certainly refrain from acting on them in many circumstances. Note, too, that the fact that I intend “acceptance” to be neutral between belief and a more behavioural response entails that I do not have to take a stand on the so-called “pragmatic encroachment” debate: I am not committed to claiming that pragmatic considerations affect whether we ought to *believe* a claim.

6 In recent work, Hanna Pickard (2013, 2017) has been developing a framework for what she calls “responsibility without blame.” We hold people responsible without blaming them when we hold them accountable for their behaviour, recognizing the degree of control they have over it and even imposing measures that might seem punitive to them, while refraining from condemnation, targeting them with hostile emotions, or rejecting them. In light of this account, it might be objected that we can avoid the negative consequences commonly associated with holding responsible without giving up on attributing responsibility. Pickard’s account faces a difficulty highlighted by Michael McKenna (2012), in response to sceptics who hold that no one should be held responsible: plausible accounts of blame can encompass even the non-punitive responses she advocates. It is open to Pickard to respond by holding that the challenge concerns how we should label certain responses to those who cause harms, rather than anything substantive: perhaps she can concede that the kinds of responses she advocates might count as blame while nevertheless insisting that some blaming responses should be avoided and others (those she identifies with holding responsible without blaming) should replace them. Pickard’s focus, however, is on certain classes of individuals and not on the broader social effects of holding responsible. She might be right about the individuals she discusses while it remains true that it would be better to avoid attributing any kind of responsibility to those who cause their own ill-health because doing so will undermine public support for effective responses to the problems at issue. If that’s right, then how we should act all things considered depends, *inter alia*, on the balance of forces at play: is it more important to support the agency of individuals held responsible or to ensure that there are effective social policies? I cannot hope to settle that issue here. Note that however that plays out, the view sketched here can cite Pickard in support of the claim that we should avoid directing harsh criticism and condemnation at those who cause the harms in question.
